# Causal effect of gallstone disease on the risk of coronary heart disease or acute myocardial infarction: a Mendelian randomization study

**DOI:** 10.1038/s41598-023-46117-9

**Published:** 2023-11-01

**Authors:** Qingan Fu, Tianzhou Shen, Qingyun Yu, Long Jiang, Renqiang Yang

**Affiliations:** Department of Cardiovascular Medicine, The Second Affiliated Hospital of Nanchang University, Nanchang, 330006 China

**Keywords:** Genetics, Diseases, Medical research, Pathogenesis

## Abstract

Gallstone disease (GSD) is thought to be associated with the risk of coronary heart disease (CHD) or acute myocardial infarction (AMI), which may be due to abnormal cholesterol metabolism. We used multiple Mendelian randomization (MR) methods based on publicly available genome-wide association study data to assess whether this association is genetically causal and to search for loci driving causality. Pooled data for GSD were obtained from FinnGen Biobank and Biobank Japan, while CHD and AMI were obtained as pooled data from the CARDIoGRAMplusC4D consortium. In this MR study, we found a significant negative causal effect of genetic susceptibility to GSD on AMI in the Finnish population, but no causal effect was found on CHD. This causal effect was not confounded by reverse causality and the same findings were obtained in the Japanese population. Furthermore, the negative causal effect of GSD on AMI risk may be driven by the rs4245791-regulated ABCG5/8 protein. In conclusion, the results of this MR study support a negative causal effect of GSD on AMI and suggest that rs4245791 is the causal driver locus of this effect, which provides new ideas and evidence for the prevention and etiologic study of AMI in patients with GSD.

## Introduction

Gallstone disease (GSD) is one of the most common diseases of the digestive system, with high costs. Approximately 10–20% of adults worldwide have gallstones; as a result, more patients with GSD are hospitalized than people with any other digestive disease^1,2^. Most patients with GSD are initially asymptomatic; as the disease progresses, GSD can lead to biliary pancreatitis, acute cholecystitis and acute cholangitis and may even be fatal^3^. Three types of gallstones are typically recognized, cholesterol stones, pigment stones and mixed stones, which are present in 90% of patients with GSD^4^. The etiology of GSD is diverse, and the formation of cholesterol gallstones is mainly due to the following causes: supersaturation of cholesterol in the bile of the gallbladder (excessive cholesterol secretion by the liver); impaired gallbladder motility; increased absorption of cholesterol from the small intestine to the liver, the intestinal microbiota and genetic factors^5,6^. These factors can accelerate production or growth of cholesterol crystals and eventually lead to stone formation.

Coronary artery disease (CHD) is the leading cause of death globally, and its prevalence has increased in recent decades. CHD also imposes a heavy economic burden of $160 billion annually worldwide^7,8^. Acute myocardial infarction (AMI) is essentially a type of CHD that results from irreversible necrosis of cardiac myocytes due to acute ischemia. Each year, approximately 1.7 million Americans are diagnosed with myocardial infarction, and more than 8 million are hospitalized due to signs and symptoms of AMI^9^. These findings undoubtedly emphasize the need to clarify the possible etiology of CHD and AMI. Altered cholesterol metabolism leads to atherosclerosis, and some studies have shown that residual cholesterol is an independent predictor of CHD or AMI^10,11^. Because cholesterol is a major component of most gallstones, there may be a link between GSD and CHD through cholesterol metabolism^12,13^. Indeed, there is evidence of a positive association between GSD and risk of CHD or AMI. However, because the mechanism of the association remains unclear and the number of available studies is small, debate regarding whether such an association exists continues^14^. Additionally, if GSD has a direct effect on CHD or AMI risk, the potential causal relationship and direction of the two diseases is uncertain owing to possible bias caused by confounding factors in previous experiments^13^.

To better address this issue, the aim of this study was to further explore the causal effect of GSD on CHD or AMI risk using multiple Mendelian randomization (MR) methods. Although the randomized controlled trial (RCT) design is the gold standard for determining causality, RCT studies have not been widely implemented in the clinical setting due to resource-intensive and severe ethical constraints. MR studies, which can address the shortcomings of traditional epidemiological studies in determining etiology, have become one of the best epidemiological tools to compensate for RCT studies; they have been widely applied in recent years to assess causality and are considered a "natural" RCT study^15^. Compared to RCT, MR has the advantages of time savings, low cost, analysis of a large amount of data and no ethical restrictions^16^. MR is a novel epidemiological approach that uses genetic variation in genome-wide association studies (GWASs) as an instrumental variable (IV) to proxy exposure factors and to infer causal relationships between exposures and outcomes. As genetic variation arises randomly during meiosis, MR can minimize the bias of results due to reverse causality and confounding factors without interference of reverse factors.

## Methods

### Study design and data source

In our study, MR analysis was used to explore a potential causal effect between a patient's genetic susceptibility to the exposure factor GSD and CHD or AMI risk outcomes. This analysis was performed on the basis of summary-level data from a GWAS of European, Finnish, and Japanese populations, for which relevant data are available from the IEU OpenGWAS project (https://gwas.mrcieu.ac.uk/). The GWAS contains thousands of sequences of human genetic variants and the effect sizes of corresponding genetic variants, which can be used to identify risk factors for disease etiology in the population. In addition, all aggregated GWAS statistics used in this study are publicly available, ethical approval was obtained in the original study, and written informed consent was provided by all participants. The analysis design strictly followed the STROBE-MR statement^17,18^, the complete STROBE-MR checklist can be found in the Supplementary Table S1.

#### GWAS of GSD

We obtained pooled data for the GSD GWAS from FinnGen Biobank with 32,894 cases and 301,383 controls of European ancestry (https://www.finngen.fi/en/access_results (r8.finngen.fi))^19^. FinnGen is a large public–private partnership that aims to collect and analyze genomic and health data for 500,000 Finnish participants, and now FinnGen is regarded as a world-class database of advances in medicine and therapeutics. The GWAS dataset for GSD in the external validation was obtained from Biobank Japan (https://biobankjp.org/en/), with a total of 487,553 samples of East Asian ancestry (26,122 cases and 461,431 controls)^20^. Diagnosis of GSD was according to International Classification of Diseases (ICD-10: K80, ICD-9:574).

#### GWAS of CHD and AMI

Summarized data on CHD GWAS were contributed by the CARDIoGRAMplusC4D and UK Biobank CardioMetabolic Consortium CHD working group, which used the UK Biobank Resource (application number 9922). The study involved 184,305 participants (60,801 CHD cases and 123,504 controls) and evaluated 9.4 million variants. AMI data, also from CARDIoGRAMplusC4D, contain 43,676 AMI cases and 128,199 control cases. Source GWAS data for CARDIoGRAMplusC4D are based on a meta-analysis of a predominantly European GWAS^21^. Data were downloaded from 'www.CARDIOGRAMPLUSC4D.ORG'. Diagnosis of CHD was according to International Classification of Diseases (ICD-10: I20, I21, I22). As mentioned in published guidelines, diagnostic criteria for AMI include the following: (1) persistent symptoms of chest pain; (2) persistent progression of ischemic signs on the electrocardiogram; and (3) increased levels of infarct-related biomarkers. Summary-level data on risk factors for AMI were obtained from the IEU OpenGWAS database. We selected several recognized risk factors for AMI, including low-density lipoprotein cholesterol (LDL-C), smoking, and hypertension^22^.

### Selection of instrumental variables

**Figure 1:**
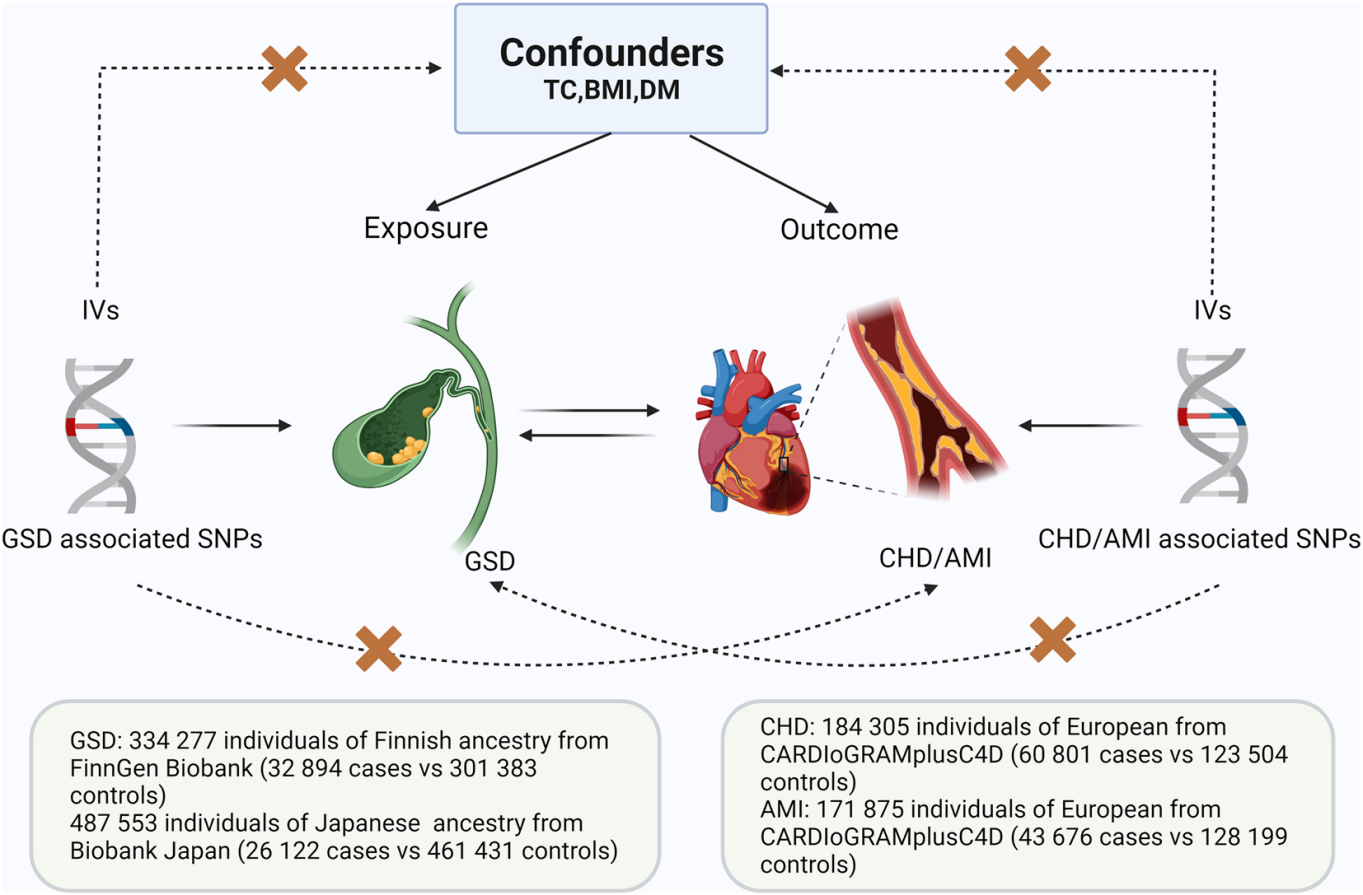
Three key principles of bidirectional Mendelian randomization study design. *GSD* gallstone disease, *CHD*, coronary heart disease, *AMI* acute myocardial infarction, *IV* instrument variant, *SNP* single-nucleotide polymorphism, *TC* total cholesterol, *BMI* body mass index, *DM* diabetes mellitus. (Created with BioRender.com).

The selection of IVs in MR analyses must conform to three principles in order to minimize experimental bias: (1) IVs must be significantly correlated with exposure factors and not directly correlated with outcomes, (2) IVs must be independent of common confounders, and (3) IVs can only influence outcomes through exposure^16^ (Fig. 1).

First, we extracted variants significantly associated with GSD (p < 5 × 10^–6^) and removed those associated with CHD or AMI (p < 5 × 10^–5^) from the corresponding GWAS database as IVs. Then, ensuring that all IVs satisfied the independence assumption, we used linkage disequilibrium to exclude ineligible IVs (threshold of r^2^ = 0.001 and window size of 10,000 kb). The threshold for r^2^ was determined by 1000 Genomes Project for populations of European ancestry. During the analysis, we removed all palindromic single-nucleotide polymorphisms (SNPs), duplicate SNPs and SNPs with missing information. To ensure that IVs are not associated with the remaining confounders of exposure and outcomes, we used the PhenoScanner website (http://www.phenoscanner.medschl.cam.ac.uk/) to find and remove SNPs corresponding to factors known to be associated with GSD, CHD or AMI (e.g., body mass index, diabetes mellitus, total cholesterol). In addition, we used the F statistic to infer the presence of weak (F-statistic < 10) IVs: F = R^2^*(N-2)/1-R^2^^23^, where R^2^ represents the variance of the IV corresponding to the exposure factor and N is the sample size (R^2^ = 2*MAF*(1-MAF)*β^2^). After calculation, the F values of all IVs were much higher than 10, which correlates strongly with exposure and guarantees the validity of SNPs^24^.

### Mendelian randomization analysis

Four MR methods were used in the study to assess the causal effect of GSD on CHD or AMI, namely, inverse variance weighting (IVW), Mendelian randomization-Egger (MR‒Egger), weighted median, and maximum likelihood^25^. Among them, IVW is the predominant MR analysis method; however, the results of IVW may be overestimated when heterogeneity is present, which is usually due to factors such as off-target genetic effects or horizontal pleiotropy^15^. And when there is heterogeneity in the MR results and no pleiotropy, the results of the weighted median method are considered to be superior to the IVW method^26^.

Although MR is a powerful technique for causal inference, several issues, including horizontal pleiotropy, epigenetic effects, and disequilibrium, may interfere with the three main assumptions of IV^27^. Funnel plots allow researchers to judge horizontal pleiotropy by visual effects, and the symmetry of the plots indicates a low probability of the presence of pleiotropy^28^. The degree of heterogeneity was assessed by calculating the Cochran-Q statistic. Furthermore, as a correction treatment for the heterogeneity of IVs, we used MR Pleiotropy RESidual Sum and Outlier (MR-PRESSO) to identify and correct for outliers in IVW linear regression and conducted sensitivity analysis to assess the reliability of the results^29^. The results of MR-PRESSO Raw can also provide complementary analysis for causality in addition to the four traditional MR analysis methods. We detected possible outliers among the selected SNPs in a multiple outlier test, and after removing the outliers using "MR-PRESSO outlier test". Furthermore, we tested whether the GSD risk estimates for CHD or AMI remained essentially the same for each SNP removed, which is called the "leave-one-out" analysis. Finally, on the basis of the traditional two-sample MR analysis, we also performed reverse MR analysis, external validation MR analysis and two-step MR analysis to make the results more comprehensive and reliable.

### Co-localization analysis

After MR analyses identified negative causality of GSD on AMI risk, we used co-localization analyses to explore loci mediating causality. Co-localization analysis is a method for estimating the posterior probability of assessing whether the same locus of genetic variation leads to a causal linkage between two phenotype or simply different causal variants that are in linkage disequilibrium with each other. The PPH4 in the posterior probability corresponds to the hypothesis that phenotype 1 (GSD) and phenotype 2 (AMI) are significantly associated with SNP loci in the genomic region and are driven by the same causally variable locus. In this study, we hypothesized that co-localization significance exists when PP.H4 > 0.80, and the greater the PP.H4 the greater the probability that the negative causality of GSD on the risk of AMI is driven by a common variant locus. LocusCompare plots were used to demonstrate the results of the co-localization analysis^30^.

### Statistical analysis

All data analyses for MR were performed in the R environment with the "TwoSampleMR" package^28^ and the "MR-PRESSO" package. The coloc R package was used to perform co-localization analysis between GSD and AMI risk^31^ (R version 4.2.2). As two rounds of analysis were performed on the dataset, statistical significance was set at P < 0.05/2 = 0.025 for this study based on Bonferroni correction.

### Institutional review board statement

All data used in this study were obtained from publicly available databases; further ethical approval was not required.

## Results

### Causal effect of GSD on risk of AMI or CHD

**Figure 2:**
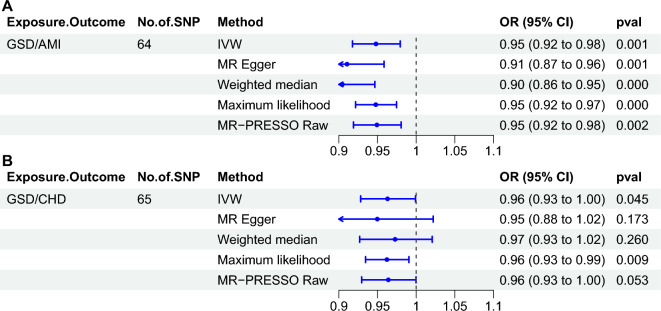
Causal effects of GSD on the risk of AMI or CHD assessed by different Mendelian randomization methods. (**A**) GSD for exposure, AMI for outcome. (**B**) GSD for exposure, CHD for outcome. *GSD* gallstone disease, *AMI* acute myocardial infarction, *IVW* inverse-variance weighted, *MR-PRESSO* MR pleiotropy residual sum and outlier, *OR* odds ratio, *CI* confidence interval.

We assessed whether there was a causal effect of GSD on AMI risk using four MR methods, which ultimately included 64 SNPs after removing confounders and outliers (Supplementary Table S1). The MR results found that all four MR methods showed significant negative causality and the MR-PRESSO Raw analyses showed the same conclusion (IVW: odds ratio (OR) = 0.95, p = 0.001; MR Egger: OR = 0.91, p = 0.001; weighted median: OR = 0.90, p = 0.000; maximum likelihood: OR = 0.95, p = 0.000; MR-PRESSO Raw: OR = 0.95, p = 0.002). In addition, there was heterogeneity in the results with no horizontal multidimensionality, and subsequent leave-one-out and funnel plot results demonstrated the robustness of our results (Supplementary Tables S2–S5, Supplementary Figs. S1–S4). The same method was subsequently used to determine the causal relationship between GSD and CHD risk, and a total of 65 SNPs were included in the analysis. The results of the MR analysis suggest that GSD may have a weak negative effect on the risk of CHD (Fig. 2), although this causal relationship was not significant, and the sensitivity analyses demonstrated the robustness of results (Supplementary Tables S6–S9, Supplementary Figs. S5–S8). The results of this MR analysis suggest that patients with GSD have a significantly reduced risk of AMI, but not a reduced risk of CHD.

### Causal effect of AMI or CHD on risk of GSD

**Figure 3:**
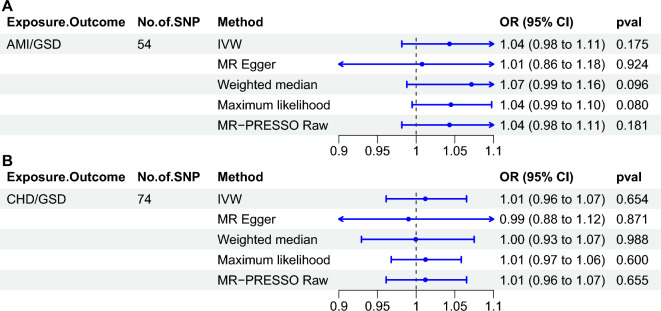
Causal effects of AMI or CHD on the risk of GSD assessed by inverse Mendelian randomization method. (**A**) AMI for exposure, GSD for outcome. (**B**) CHD for exposure, GSD for outcome. *GSD* gallstone disease, *AMI* acute myocardial infarction, *IVW* inverse-variance weighted, *MR-PRESSO* MR pleiotropy residual sum and outlier, *OR* odds ratio, *CI* confidence interval.

To exclude the possibility that the negative causal effect of GSD on AMI risk could be influenced by reverse causality, we further performed a reverse MR analysis. In the analysis, 54 and 74 SNPs were included for AMI or CHD as exposure and GSD as outcome, respectively (Supplementary Tables S10–S13). However, neither AMI nor CHD was observed to have a significant causal effect on GSD, each MR method showed the same results (Fig. 3). Cochran-Q test, Funnel plots, leave-one-out method and F-values greater than 10 for all SNPs proved that our results are reliable (Supplementary Figs. S9–S16). That is, there is no causal effect of AMI or CHD on the risk of GSD, and the causal relationship between GSD and decreased risk of AMI is not disturbed by reverse causal effects.

### External validation of another GSD dataset

**Figure 4:**
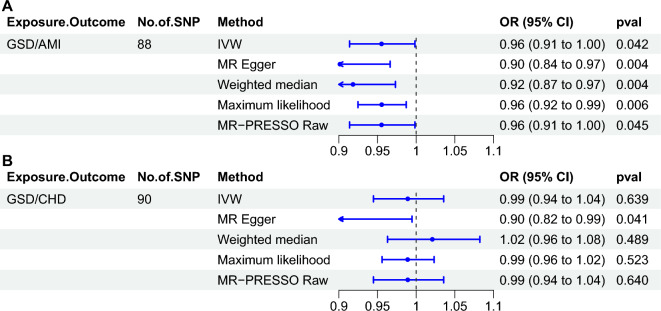
Causal effects of GSD on the risk of AMI or CHD assessed by inverse Mendelian randomization method in external validation. (**A**) GSD for exposure, AMI for outcome. (**B**) GSD for exposure, CHD for outcome. *GSD* gallstone disease, *AMI* acute myocardial infarction, *IVW* inverse-variance weighted, *MR-PRESSO* MR pleiotropy residual sum and outlier, *OR* odds ratio, *CI* confidence interval.

We also wished to explore whether the negative causal effect of GSD on the risk of AMI persists in ethnic groups other than those of Finnish origin, in order to increase the generalizability of the findings to various populations. As a result, another GSD database of East Asian ancestry with a predominantly Japanese population was included for externally validated MR analysis. In exploring the causal effect of GSD on AMI risk, the results of the MR analyses again demonstrated a significantly negative causal effect (Fig. 4). Similarly, we found that a significant causal relationship between GSD and CHD was still not found in the new GWAS data, which is consistent with the results in the Finnish population (Supplementary Tables S14–S17, Supplementary Figs. S17–S24). The fact that the same conclusions were obtained in an external validation further provides strong backup for our study and suggests that the results may be applicable to a wider population.

### Mediation by LDL-C, smoking and hypertension

Multiple risk factors have the potential to contribute to the development of AMI, so is the causal effect of GSD in reducing the risk of AMI mediated through these risk factors? We selected 3 traditional risk factors for AMI, such as LDL-C, smoking and hypertension, for two-step MR analysis. The results of the study found no causal association between GSD and the 3 mediators, although LDL-C, smoking and hypertension all demonstrated significant causal effects on AMI (Supplementary Tables S18). This suggests that the causal effect between GSD and reduced risk of AMI is not mediated through LDL-C, smoking and hypertension, further exploration of the specific causal drivers is still needed.

### Co-localization analysis

It has been suggested that the significance of MR results may be derived from SNPs in a state of linkage disequilibrium and that the associated SNPs for exposure and outcome are different causal SNPs, which may lead to false-positive inferred results^32^.

**Figure 5:**
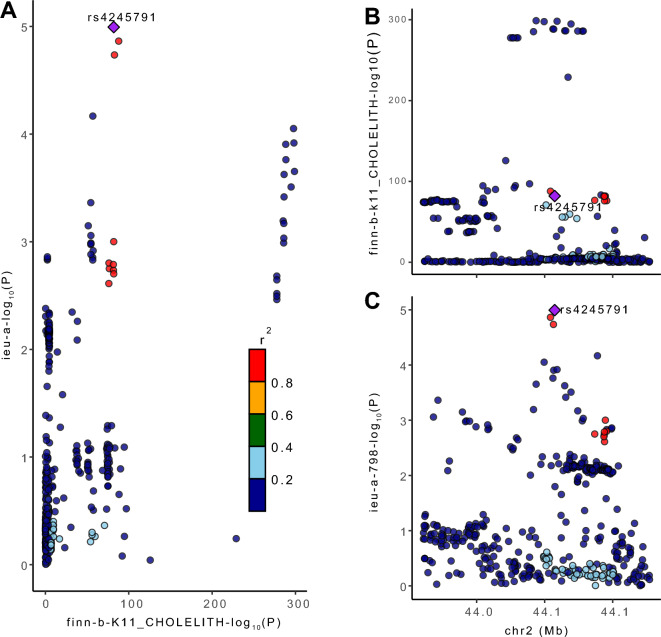
The LocusCompare plot marks the driver causal loci and distinguishes false positive genes. (**A**) The distribution of SNPs − log10(p) in the GWAS of GSD and AMI is depicted, with rs4245791 viewed as a co-localized locus driving causality. (**B**) The − log10(p) distribution of rs4245791 in the GWAS of GSD and its location on the chromosome are shown. (**C**) The − log10(p) distribution of rs4245791 in the GWAS of AMI and its location on the chromosome are shown. The Y-axis represents the − log10(p) value of the SNP in the GWAS data, with higher positions representing smaller p-values and more significant correlations. The x-axis of the graph represents the position of the SNP on the chromosome.

Co-localization analysis can be used to explore whether exposure and outcome share the same causal SNP locus, and there is evidence that proteins tested by MR and co-localization would be more valuable therapeutic targets^31^. We therefore performed co-localization analyses between GSD and GWAS data from AMI to assess potential confounders due to linkage disequilibrium and to find specific causal driver SNPs. Among the results, it was found that rs4245791 had the largest PP.H4 value (PP.H4 = 0.87, PP.H4 > 0.80) and the smallest sum of p-values for the correlation between the two phenotypes (Fig. 5, Supplementary Tables S19–S21), which indicated that rs4245791 had strong correlation with the two phenotypes and was the most potent locus for driving causality between the phenotypes. The ABCG8 protein regulated by rs4245791 could explain the negative causal effect of GSD on the risk of AMI.

## Discussion

In the present study, MR analysis showed a significant negative causal effect of GSD on AMI risk but did not reveal a causal relationship between GSD and CHD risk. This finding was reconfirmed in an external validation conducted on a population of Japanese ancestry. We then performed a reverse MR study to exclude the existence of reverse causality. Finally, in co-localization analysis, rs4245791 was found to be the core locus that may mediate the negative causal effect of GSD on AMI, which provides strong evidentiary support for the etiological explanation of the decreased risk of AMI in GSD patients.

Many existing studies support the conclusion that patients with GSD have higher risk of CHD, a finding that also applies to AMI. As early as 1985, the Framingham study found a possible association between GSD and CHD, and researchers recommended GSD as a risk factor for CHD in men^33^. Subsequent studies found that GSD is an independent predictor of atherosclerosis progression, i.e., GSD is indeed significantly associated with increased risk of CHD and AMI^13,14^. Furthermore, in the cohort established by Daniel et al., GSD was found to increase risk of cerebrovascular disease and peripheral arterial disease^34^. However, different results were found in the study by Zhang et al., who reported that the presence of GSD improves prognosis of acute coronary syndromes; in another cohort study with a 10-year follow-up in a British population, no association between GSD and CHD risk was found, which seems to be consistent with our findings^35,36^. To date, the number of prospective studies on GSD and risk of CHD or AMI remains small, with conflicting findings and traditional retrospective clinical studies failing to accurately reveal the causal impact of GSD on risk of CHD or AMI^37^.

The positive correlation exhibited by GSD with CHD or AMI risk in most studies may be due to a large number of common risk factors. For example, obesity, diabetes mellitus and hypercholesterolemia, which can lead to crystallization of supersaturated cholesterol in bile to form gallstones, increase cholesterol deposition in the lining and accelerate the AS process, respectively, do not show an intrinsic causal influence^38^. Insulin-like growth factor-1 (IGF-1) is a mitogenic factor that promotes tissue repair and cell proliferation, and there is evidence that IGF-1 serum levels may be associated with susceptibility to gallstones^39,40^. In addition, IGF-1 deficiency is an important factor in development of atherosclerosis, and IGF-1 levels may be negatively associated with cholesterol deposition and plaque formation^41^. However, at present, this association is not sufficient to prove that IGF-1 is a causal influence of GSD on risk of CHD or AMI^42^. Similarly, metabolites of the gut microbiota and inflammation have been suggested to link GSD to CHD or AMI risk, but whether they are causal influences remains to be demonstrated by intrinsic mechanisms^43–45^. In our study, in order to investigate whether the causal relationship between GSD and AMI is caused by traditional cardiovascular risk factors, we performed two-step MR analysis with LDL-C, smoke, and hypertension as mediators. However, the results suggest that the causal relationship is not independently mediated by the above factors, and the specific causal drivers may be otherwise.

A number of potential mechanisms may provide an explanation for our conclusions. In the results of co-localization analysis we found that rs4245791 is a key locus mediating the causal relationship between GSD and AMI, and it has been shown that rs4245791 is strongly correlated with ABCG8^46^. The D19H polymorphism in ABCG8 is by far the most prominent genetic determinant of GSD, GWAS data also showed that mutations in some loci of ABCG5/8 were strongly associated with GSD^47^. The link between GSD and cardiovascular disease is thought to arise through ABCG5/8, a transporter protein that promotes the secretion of cholesterol and phytosterols into the bile^34,48^. In animal studies, researchers found that high expression of ABCG5/8 attenuated hypercholesterolemia in Ldlr−/− mice, which in turn significantly ameliorated atherosclerotic lesions in the aorta^49^. In humans, gain-of-function due to variants in the ABCG5/8 gene has been shown to increase the amount of cholesterol discharged from hepatocytes to the gallbladder, thereby directly increasing the lithogenicity of bile and indirectly decreasing LDL-C concentrations and the risk of AMI^50^. The above studies provide strong evidence to support our conclusion that the negative causality of GSD on AMI risk is mediated by ABCG5/8 gain of function, which makes the results more reliable and valuable. miRNA-223, on the other hand, has been found by researchers to directly target the inhibition of ABCG5/8 to reduce the occurrence of GSD, but whether it will inversely lead to an increased risk of AMI is not conclusive^51^. In addition, animal studies have shown that deletion of the ABCG5/8 gene does not eliminate hepatic cholesterol secretion to the gallbladder and that other ABCG5/8 nondependent pathways involved in hepatic cholesterol secretion exist^52,53^. This implies that enhanced ABCG5/8 expression in GSD patients reduces serum cholesterol levels, but only to a limited extent. In general, AMI patients have higher cholesterol levels than CHD patients, and some cholesterol-lowering effect of ABCG5/8 may be sufficient to significantly reduce risk of AMI, an acute CHD type disease, but not sufficient to cause a significant change in overall CHD risk. This explains the significant negative causal effect of GSD on AMI risk found in our study, though this negative effect was present but not significant for CHD risk. In conclusion, ABCG5/8 can still be regarded as a potential drug target for lipid-lowering therapy, and its gain-of-function regulatory mechanisms and ameliorative effects on AMI risk will continue to be deeply investigated in future studies.

The strengths of our study are as follows. First, our study is the first MR study to analyze the causal effect of GSD on CHD or AMI. We included GWAS datasets from multiple populations in our study and also performed external validation as well as reverse MR analysis. This makes our findings more comprehensive and reliable and can be applied to a wider range of populations. Second, MR‒Egger regression results showed no cross-sectional polymorphisms in the IVs; after removing possible heterozygous loci, both the leave-one-out method and the funnel plot showed our conclusions to be robust and plausible. Finally, co-localization analysis was used by us to explore the specific sites mediating causality, which allowed for more in-depth studies. The results provide support for the causal relationship observed by MR analysis at the mechanistic level and provide precise targets for researchers to modulate this relationship.

However, there are also limitations that cannot be ignored. Initially, the study identified a key role for rs4245791 in reducing the risk of AMI in GSD patients, but we did not further explore its potential as a therapeutic target nor did animal experiments validate the conclusions. In addition, although the database used in this study included two major populations of Finnish and East Asian ancestry, this is still not representative of the rest of the global population, and the applicability of the findings to ethnic groups other than those of Finnish and Japanese origin needs to be further explored. Ultimately, sex and age factors may also influence the causal relationship between exposure and outcome. Further analysis of sex-age stratified cohorts could be conducted, as data allow, to explore the impact.

## Conclusions

Overall, the results of this MR study found a protective causal effect of GSD on AMI, suggesting that patients with GSD may have a lower risk of AMI but no benefit on the risk of CHD. Co-localization analyses further identified a key role for rs4245791 and its regulated ABCG5/8 proteins in driving the causal effect of GSD on AMI, providing strong evidence to support the conclusions. We believe that the present study makes a valuable contribution to the field by providing a novel perspective and deeper explanation of the correlation between GSD and AMI. More prospective studies as well as basic experiments are needed in future studies to continue to explore the mechanism of association between GSD and AMI and the possible clinical benefits of targeting ABCG5/8.

### Supplementary Information

Supplementary Figures.

Supplementary Table S1.

Supplementary Tables.

## Data Availability

The datasets analyzed during the current study are available in FinnGen Biobank (https://www.finngen.fi/en/access_results(r8.finngen.fi)), Biobank Japan (https://biobankjp.org/en/) and CARDIoGRAMplusC4D (www.CARDIOGRAMPLUSC4D.ORG). All data can be retrieved from the IEU OpenGWAS (https://gwas.mrcieu.ac.uk/), further inquiries can be directed to the corresponding authors.

## References

[1] Gutt C, Schläfer S, Lammert F (2020). The treatment of gallstone disease. Dtsch. Arztebl. Int..

[2] EASL Clinical Practice Guidelines on the prevention (2016). diagnosis and treatment of gallstones. J. Hepatol..

[3] Tanaka H (2018). Claudin-3 regulates bile canalicular paracellular barrier and cholesterol gallstone core formation in mice. J. Hepatol..

[4] Qiao T (2013). The systematic classification of gallbladder stones. PLoS One.

[5] Grigor'eva IN, Romanova TI (2020). Gallstone disease and microbiome. Microorganisms.

[6] Wang HH, Portincasa P, Afdhal NH, Wang DQ (2010). Lith genes and genetic analysis of cholesterol gallstone formation. Gastroenterol. Clin. North Am..

[7] Lloyd-Jones D (2010). Executive summary: Heart disease and stroke statistics–2010 update: A report from the American Heart Association. CIRCULATION.

[8] Tsao CW (2022). Heart disease and stroke statistics-2022 update: A report from the American Heart Association. Circulation.

[9] DeFilippis AP (2019). Assessment and treatment of patients with type 2 myocardial infarction and acute nonischemic myocardial injury. Circulation.

[10] Aguilar-Ballester M, Herrero-Cervera A, Vinué Á, Martínez-Hervás S, González-Navarro H (2020). Impact of cholesterol metabolism in immune cell function and atherosclerosis. Nutrients.

[11] Xiang QY (2020). Comparison of remnant cholesterol levels estimated by calculated and measured LDL-C levels in Chinese patients with coronary heart disease. Clin. Chim. Acta.

[12] Olaiya MT, Chiou HY, Jeng JS, Lien LM, Hsieh FI (2013). Significantly increased risk of cardiovascular disease among patients with gallstone disease: A population-based cohort study. PLoS One.

[13] Zheng Y (2016). Gallstones and risk of coronary heart disease: Prospective analysis of 270 000 men and women from 3 US cohorts and meta-analysis. Arterioscler. Thromb. Vasc. Biol..

[14] Yu KJ (2017). Gallstone disease is associated with arterial stiffness progression. Hypertens. Res..

[15] Davey Smith G, Hemani G (2014). Mendelian randomization: Genetic anchors for causal inference in epidemiological studies. Hum. Mol. Genet..

[16] Davies NM, Holmes MV, Davey Smith G (2018). Reading Mendelian randomisation studies: A guide, glossary, and checklist for clinicians. Bmj.

[17] Smith GD, Davies NM, Dimou N, Egger M, Gallo V, Golub R, Higgins JP, Langenberg C, Loder EW, Richards JB, Richmond RC, Skrivankova VW, Swanson SA, Timpson NJ, Tybjaerg-Hansen A, VanderWeele TJ, Woolf BA, Yarmolinsky J (2019). STROBE-MR: Guidelines for strengthening the reporting of Mendelian randomization studies. PeerJ Preprints.

[18] Skrivankova VW (2021). Strengthening the reporting of observational studies in epidemiology using mendelian randomisation (STROBE-MR): Explanation and elaboration. Bmj.

[19] Kurki MI (2023). FinnGen provides genetic insights from a well-phenotyped isolated population. Nature.

[20] Sakaue S (2021). A cross-population atlas of genetic associations for 220 human phenotypes. Nat. Genet..

[21] Nikpay M (2015). A comprehensive 1,000 Genomes-based genome-wide association meta-analysis of coronary artery disease. Nat Genet..

[22] Reed GW, Rossi JE, Cannon CP (2017). Acute myocardial infarction. Lancet.

[23] Pierce BL, Ahsan H, Vanderweele TJ (2011). Power and instrument strength requirements for Mendelian randomization studies using multiple genetic variants. Int. J. Epidemiol..

[24] Burgess S, Small DS, Thompson SG (2017). A review of instrumental variable estimators for Mendelian randomization. Stat. Methods Med. Res..

[25] Bowden J (2018). Improving the visualization, interpretation and analysis of two-sample summary data Mendelian randomization via the Radial plot and Radial regression. Int. J. Epidemiol..

[26] Burgess S, Thompson SG (2017). Interpreting findings from Mendelian randomization using the MR-Egger method. Eur. J. Epidemiol..

[27] Wang B (2022). Using genetic instruments to estimate the causal effect of hormonal reproductive factors on osteoarthritis. Front Public Health.

[28] Hemani G (2018). The MR-Base platform supports systematic causal inference across the human phenome. Elife.

[29] Verbanck M, Chen CY, Neale B, Do R (2018). Detection of widespread horizontal pleiotropy in causal relationships inferred from Mendelian randomization between complex traits and diseases. Nat. Genet..

[30] Liu B, Gloudemans MJ, Rao AS, Ingelsson E, Montgomery SB (2019). Abundant associations with gene expression complicate GWAS follow-up. Nat. Genet..

[31] Giambartolomei C (2014). Bayesian test for colocalisation between pairs of genetic association studies using summary statistics. PLoS Genet..

[32] Hemani G, Bowden J, Davey Smith G (2018). Evaluating the potential role of pleiotropy in Mendelian randomization studies. Hum. Mol. Genet..

[33] Bortnichak EA (1985). The association between cholesterol cholelithiasis and coronary heart disease in Framingham, Massachusetts. Am. J. Epidemiol..

[34] Shabanzadeh DM, Skaaby T, Sørensen LT, Jørgensen T (2017). Screen-detected gallstone disease and cardiovascular disease. Eur. J. Epidemiol..

[35] Su W, Zhu JG, Li WP, Chen H, Li HW (2022). Gallstone disease and the risk of cardiac mortality in patients with acute coronary syndrome. Front. Cardiovasc. Med..

[36] Khan HN, Harrison M, Bassett EE, Bates T (2009). A 10-year follow-up of a longitudinal study of gallstone prevalence at necropsy in South East England. Dig. Dis. Sci..

[37] Grimes DA, Schulz KF (2002). Descriptive studies: What they can and cannot do. Lancet.

[38] Méndez-Sánchez N (2005). Metabolic syndrome as a risk factor for gallstone disease. World J. Gastroenterol..

[39] Twickler MT, Cramer MJ, van Erpecum KJ (2005). Insulin-like growth factor-1: A common metabolic pathway in the origin of both gallstones and coronary heart disease. Am. J. Gastroenterol..

[40] Moschetta A (2004). Effects of growth hormone deficiency and recombinant growth hormone therapy on postprandial gallbladder motility and cholecystokinin release. Dig. Dis. Sci..

[41] Juul A, Scheike T, Davidsen M, Gyllenborg J, Jørgensen T (2002). Low serum insulin-like growth factor I is associated with increased risk of ischemic heart disease: A population-based case-control study. Circulation.

[42] Méndez-Sánchez N (2008). Gallstones are associated with carotid atherosclerosis. Liver Int..

[43] Geetha A (2002). Evidence for oxidative stress in the gall bladder mucosa of gall stone patients. J. Biochem. Mol. Biol. Biophys..

[44] Ridker PM (2016). From C-reactive protein to interleukin-6 to interleukin-1: Moving upstream to identify novel targets for atheroprotection. Circ. Res..

[45] Beukers NG, van der Heijden GJ, van Wijk AJ, Loos BG (2017). Periodontitis is an independent risk indicator for atherosclerotic cardiovascular diseases among 60 174 participants in a large dental school in the Netherlands. J. Epidemiol. Community Health.

[46] Joshi AD (2016). Four susceptibility loci for gallstone disease identified in a meta-analysis of genome-wide association studies. Gastroenterology.

[47] von Kampen O (2013). Genetic and functional identification of the likely causative variant for cholesterol gallstone disease at the ABCG5/8 lithogenic locus. Hepatology.

[48] Buch S (2007). A genome-wide association scan identifies the hepatic cholesterol transporter ABCG8 as a susceptibility factor for human gallstone disease. Nat. Genet..

[49] Wilund KR, Yu L, Xu F, Hobbs HH, Cohen JC (2004). High-level expression of ABCG5 and ABCG8 attenuates diet-induced hypercholesterolemia and atherosclerosis in Ldlr-/- mice. J. Lipid Res..

[50] Hancock-Cerutti W, Rader DJ (2014). Opposing effects of ABCG5/8 function on myocardial infarction and gallstone disease. J. Am. Coll. Cardiol..

[51] Zhao F (2021). miRNA-223 suppresses mouse gallstone formation by targeting key transporters in hepatobiliary cholesterol secretion pathway. Int. J. Biol. Sci..

[52] Wang HH, Patel SB, Carey MC, Wang DQ (2007). Quantifying anomalous intestinal sterol uptake, lymphatic transport, and biliary secretion in Abcg8(-/-) mice. Hepatology.

[53] Wang HH, Liu M, Portincasa P, Wang DQ (2020). Recent advances in the critical role of the sterol efflux transporters ABCG5/G8 in health and disease. Adv. Exp. Med. Biol..

