# Supplementary material for: Causal effect of gallstone disease on the risk of coronary heart disease or acute myocardial infarction: a Mendelian randomization study
Source: Sci Rep. 2023 Nov 1;13:18807. doi: 10.1038/s41598-023-46117-9 (PMC10620410; doi:10.1038/s41598-023-46117-9)

**Supplementary Figure Legends**

**Figure S1.** The causal effect of GSD on AMI risk before and after removal of confounders and outliers was assessed by different MR methods.

**Figure S2.** Multiple MR tests show effect values for SNPs in GSD and AMI associations after removal of outliers and confounders.

**Figure S3.** Leave-one-out analysis for GSD with AMI after removal of outliers and confounders.

**Figure S4.** Funnel plot of the effect of GSD on AMI risk after removal of outliers and confounders.

**Figure S5.** The causal effect of GSD on CHD risk before and after removal of confounders and outliers was assessed by different MR methods.

**Figure S6.** Multiple MR tests show effect values for SNPs in GSD and CHD associations after removal of outliers and confounders.

**Figure S7.** Leave-one-out analysis for GSD with CHD after removal of outliers and confounders.

**Figure S8.** Funnel plot of the effect of GSD on CHD risk after removal of outliers and confounders.

**Figure S9.** The causal effect of AMI on GSD risk after removal of confounders and outliers was assessed by different MR methods.

**Figure S10.** Multiple MR tests show effect values for SNPs in AMI and GSD associations after removal of outliers and confounders.

**Figure S11.** Leave-one-out analysis for AMI with GSD after removal of outliers and confounders.

**Figure S12.** Funnel plot of the effect of AMI on GSD risk after removal of outliers and confounders.

**Figure S13.** The causal effect of CHD on GSD risk after removal of confounders and outliers was assessed by different MR methods.

**Figure S14.** Multiple MR tests show effect values for SNPs in CHD and GSD associations after removal of outliers and confounders.

**Figure S15.** Leave-one-out analysis for CHD with GSD after removal of outliers and confounders.

**Figure S16.** Funnel plot of the effect of CHD on GSD risk after removal of outliers and confounders.

**Figure S17.** Causal effect of GSD on AMI risk after removal of confounders and outliers in externally validated MR analysis.

**Figure S18.** Multiple MR tests showed effect values for GSD and AMI-related SNPs in external validation after removing outliers and confounders.

**Figure S19.** Leave-one-out analysis for GSD with AMI in external validation after removal of outliers and confounders.

**Figure S20.** Funnel plot of the effect of GSD on AMI risk in external validation after removal of outliers and confounders.

**Figure S21.** Causal effect of GSD on CHD risk after removal of confounders and outliers in externally validated MR analysis.

**Figure S22.** Multiple MR tests showed effect values for GSD and CHD-related SNPs in external validation after removing outliers and confounders.

**Figure S23.** Leave-one-out analysis for GSD with CHD in external validation after removal of outliers and confounders.

**Figure S24.** Funnel plot of the effect of GSD on CHD risk in external validation after removal of outliers and confounders.

**Figure S1**


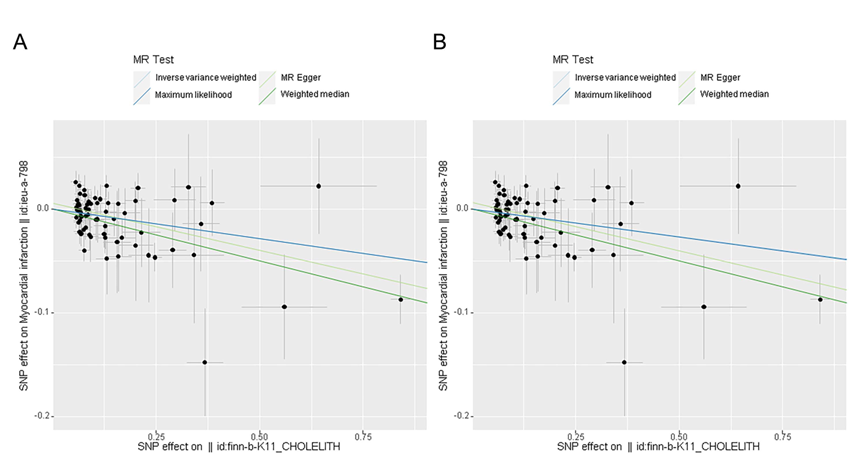


**Figure S2**


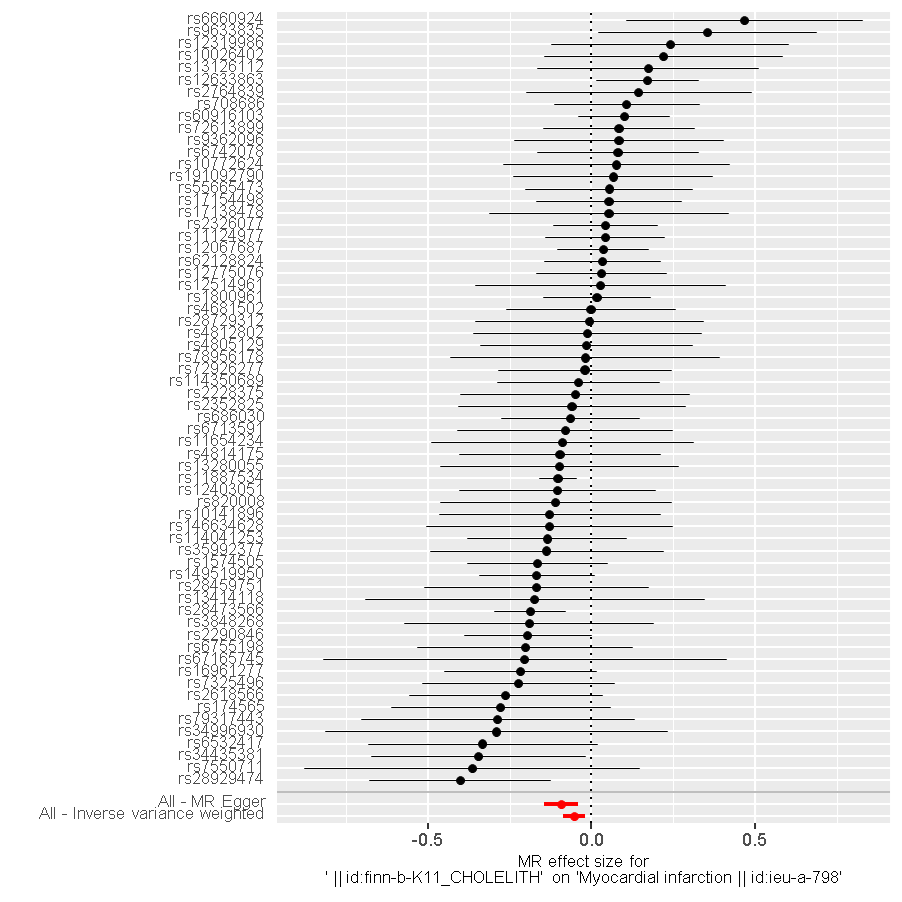


**Figure S3**


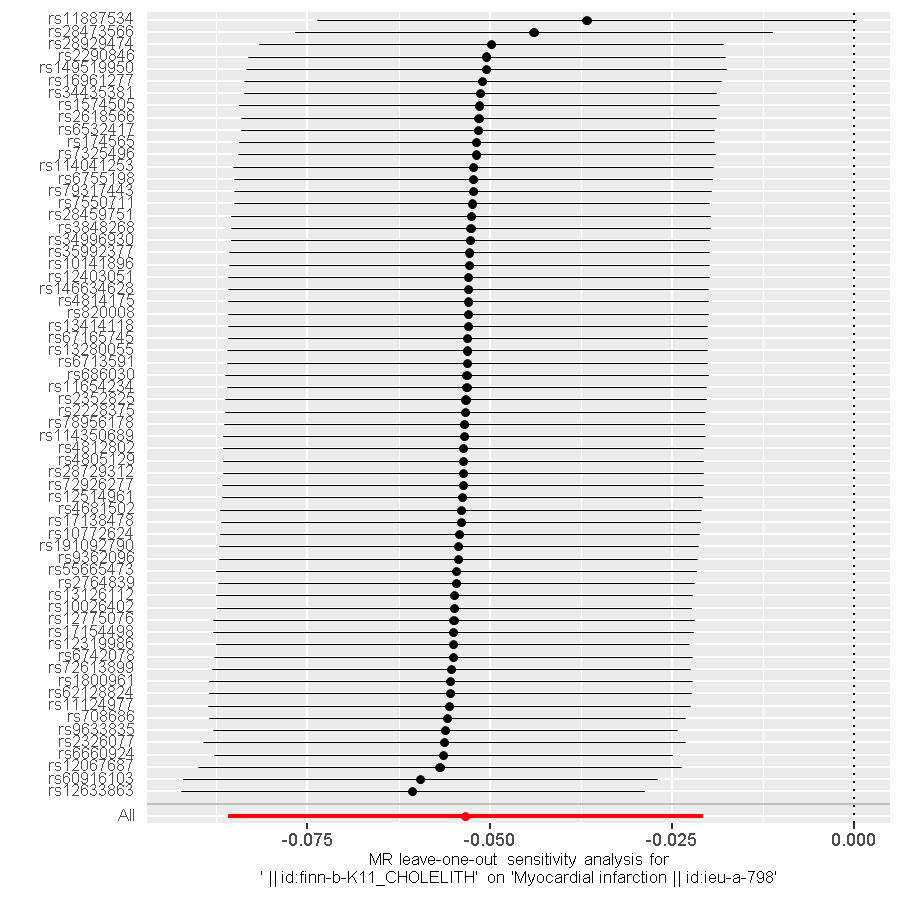


**Figure S4**


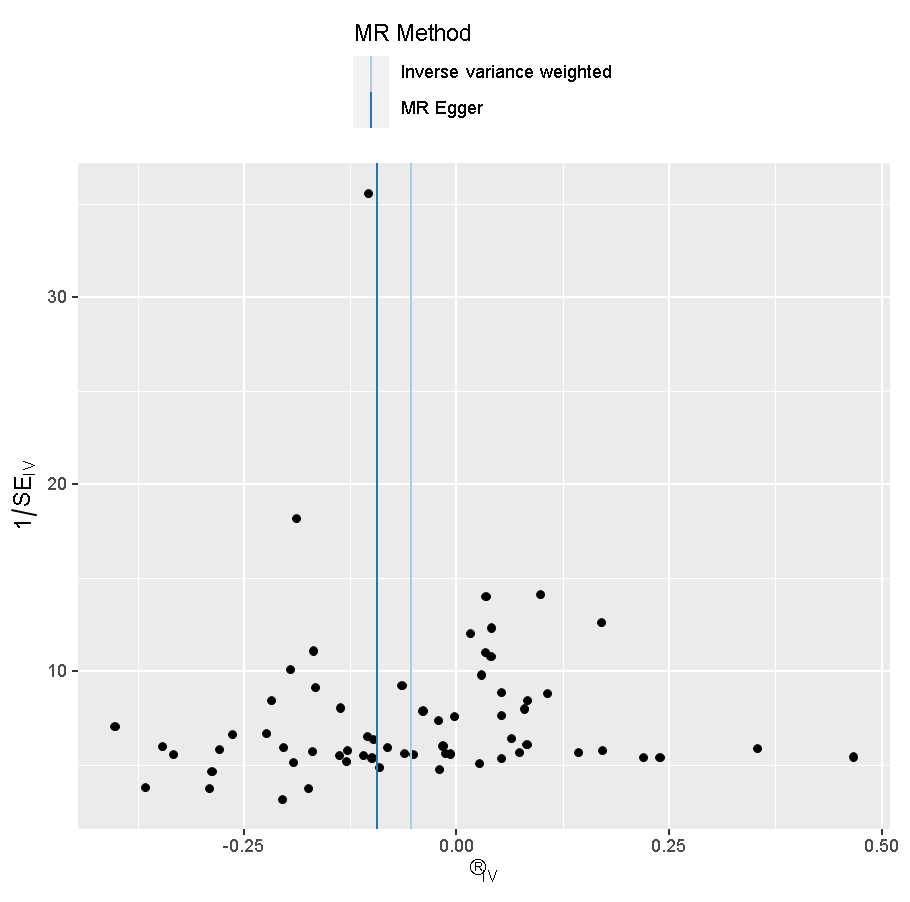


**Figure S5**


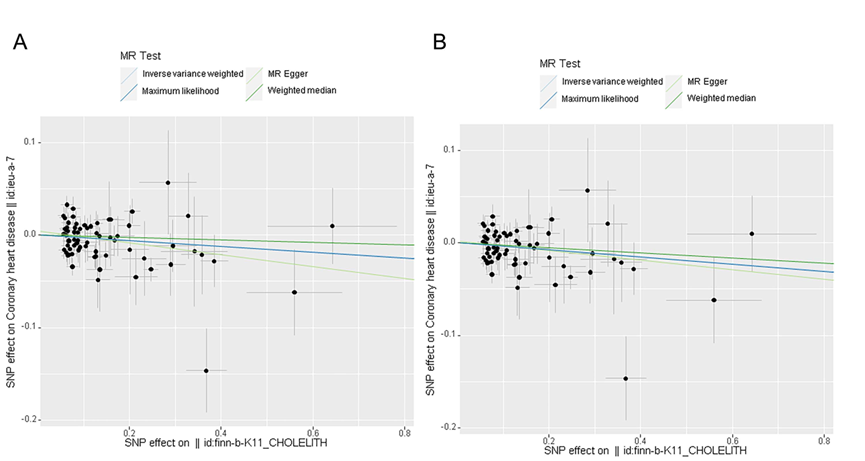


**Figure S6**


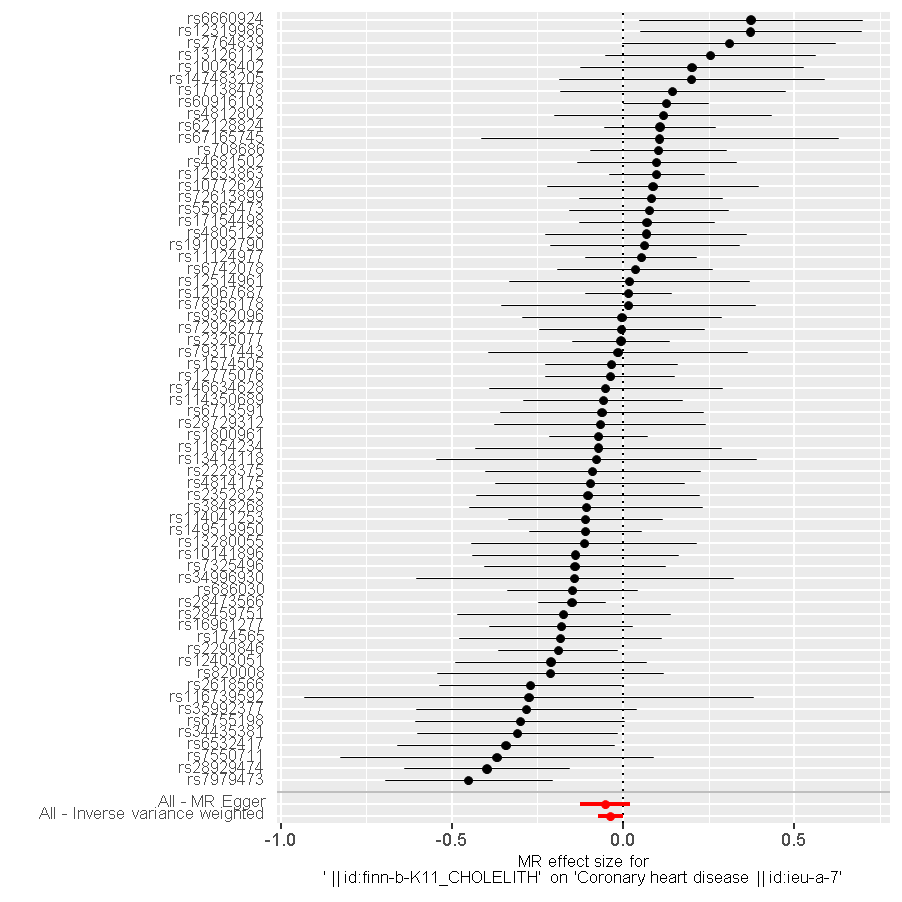


**Figure S7**


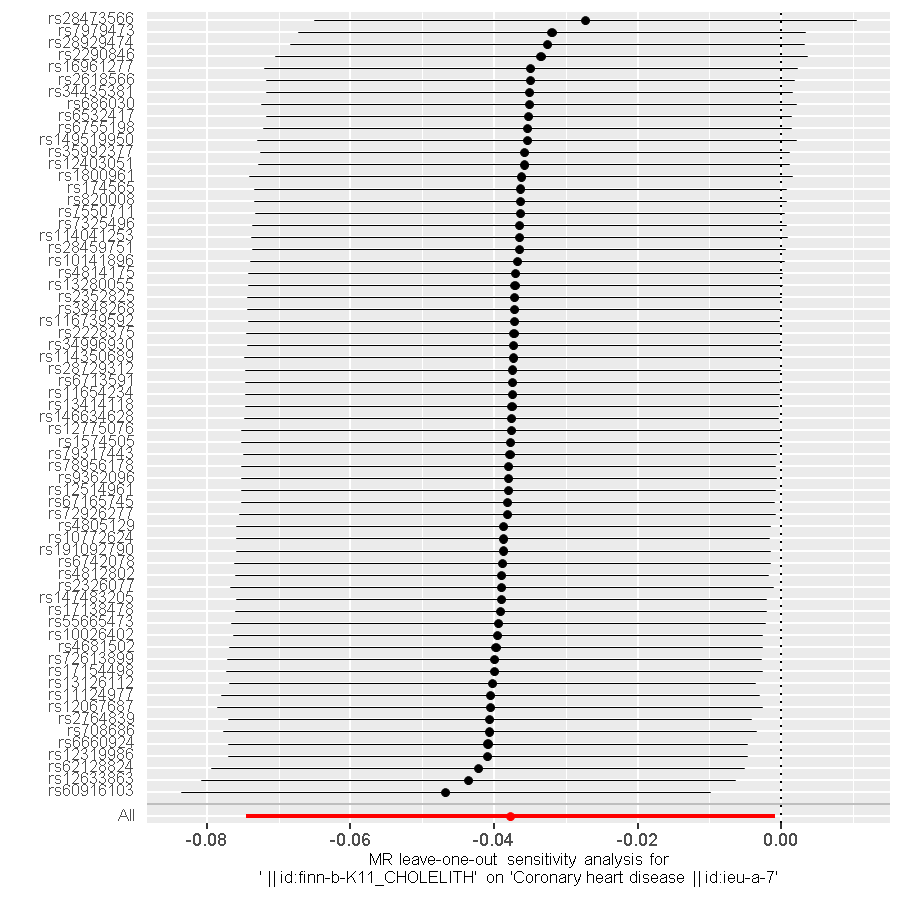


**Figure S8**


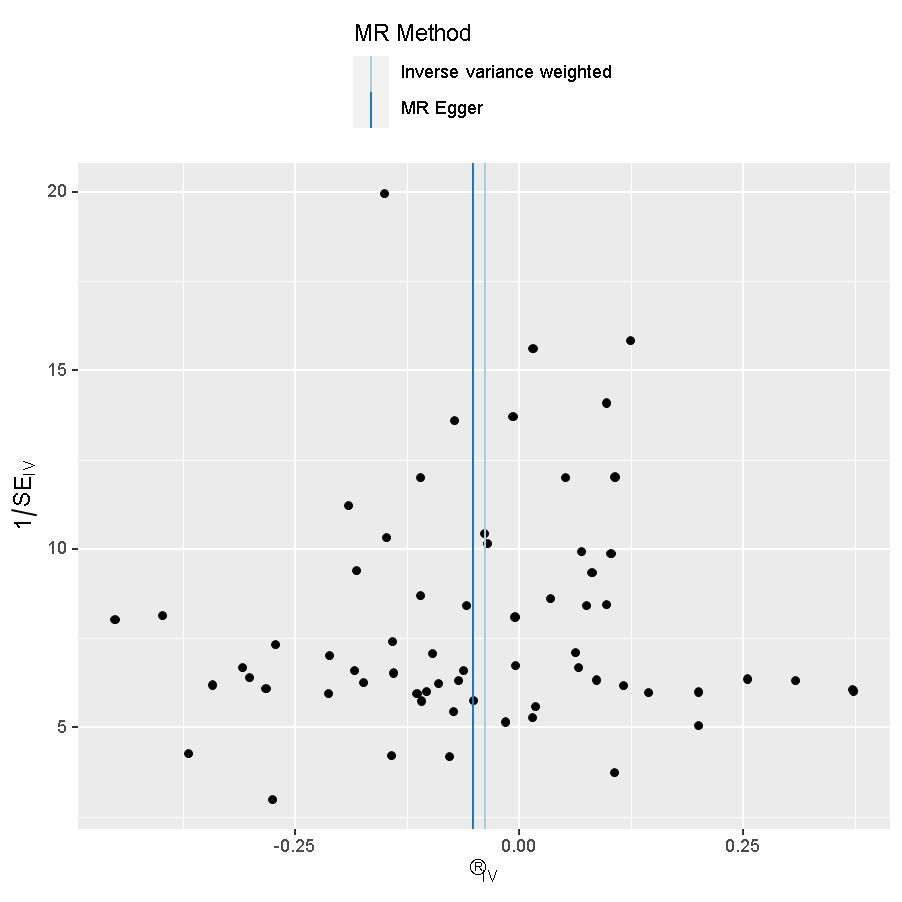


**Figure S9**


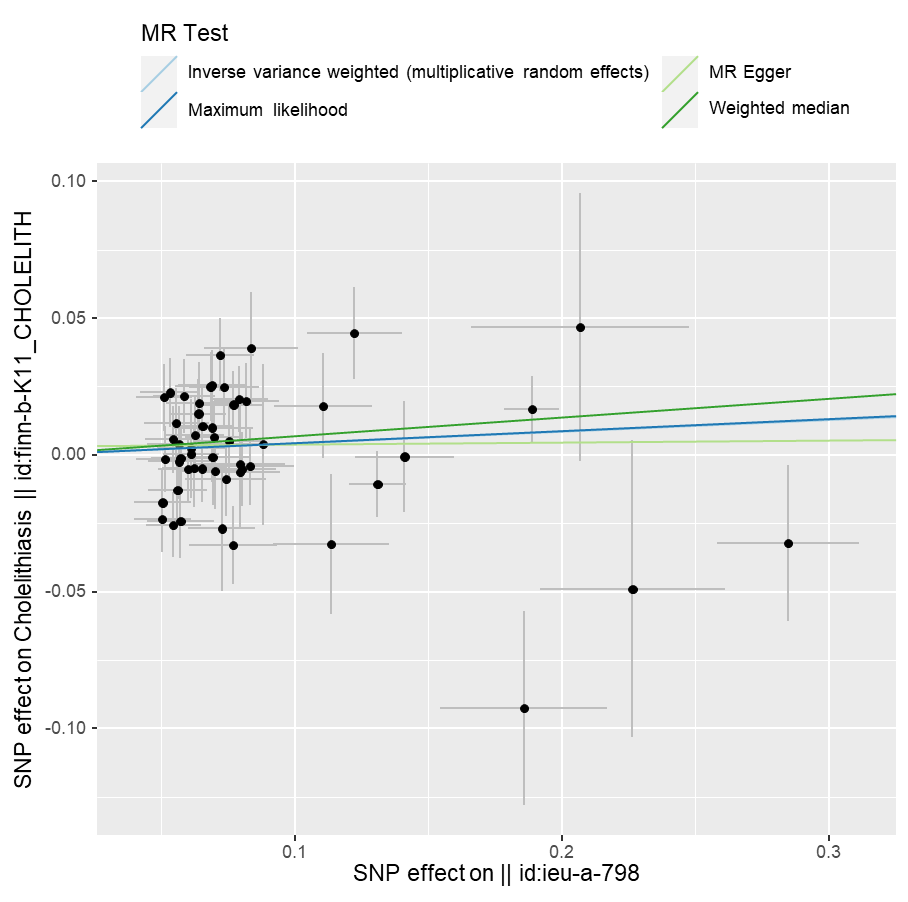


**Figure S10**

All - Inverse variance weighted

All - MR Egger

rs41290120

rs12118721

rs6761276

rs15052

rs7098414

rs2681472

rs1500187

rs80302977

rs2280003

rs1510226

rs1133773

rs10455872

rs8039305

rs72743461

rs9349379

rs1004467

rs12743267

rs8045120

rs4773141

rs111533129

rs11099493

rs16986953

rs14228

rs9595963

rs17612742

rs72934535

rs2505083

rs9486719

rs1873197

rs113113862

rs10818583

rs4977574

rs1870634

rs2836631

rs2327426

rs17282078

rs2019090

rs9970807

rs10857147

rs13291603

rs7173743

rs653178

rs35700460

rs1332329

rs2286198

rs72689147

rs28451064

rs4796663

rs11125089

rs11556924

rs748431

rs2760740

rs34821320

rs10947786

-1.0

-0.5

0.0

0.5

1.0

MR effect size for

'|| id:ieu-a-798' on 'Cholelithiasis || id:finn-b-K11_CHOLELITH'

**Figure S11**

All

rs28451064

rs10947786

rs4977574

rs4796663

rs11556924

rs1332329

rs653178

rs72689147

rs35700460

rs34821320

rs2760740

rs748431

rs11125089

rs7173743

rs2286198

rs9970807

rs13291603

rs10857147

rs2019090

rs2327426

rs1870634

rs2836631

rs17282078

rs113113862

rs10818583

rs1873197

rs9486719

rs17612742

rs2505083

rs16986953

rs14228

rs9595963

rs11099493

rs1004467

rs72934535

rs12743267

rs111533129

rs8039305

rs8045120

rs72743461

rs4773141

rs2681472

rs1510226

rs1133773

rs2280003

rs80302977

rs1500187

rs7098414

rs6761276

rs12118721

rs15052

rs41290120

rs9349379

rs10455872

0.00

0.04

0.08

0.12

MR leave-one-out sensitivity analysis for

'|| id:ieu-a-798' on 'Cholelithiasis || id:finn-b-K11_CHOLELITH'

**Figure S12**

4

8

12

16

-0.50

-0.25

0.00

0.25

0.50

β

I

V

1

S

E

I

V

MR Method

Inverse variance weighted

MR Egger

**Figure S13**


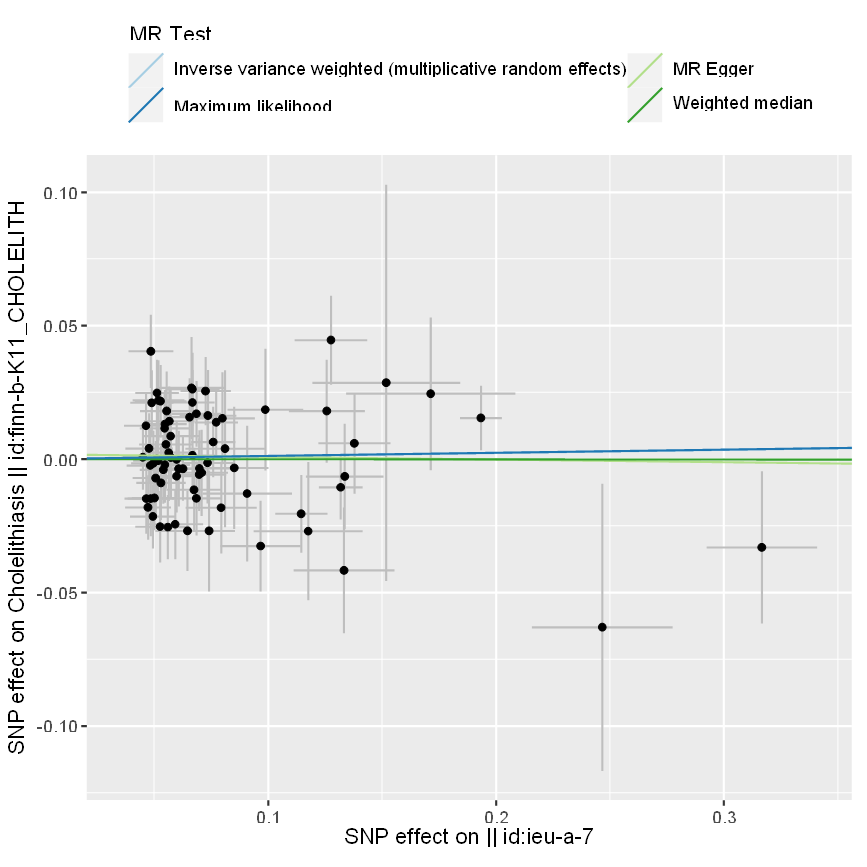


**Figure S14**

All - Inverse variance weighted

All - MR Egger

rs7082705

rs6689306

rs28470722

rs35465346

rs7188857

rs2789422

rs2681472

rs8042271

rs8104311

rs3918226

rs10071096

rs1924981

rs10080815

rs58045791

rs139833223

rs11838776

rs7528419

rs515135

rs17411031

rs11170820

rs10841443

rs2521501

rs55730499

rs56062135

rs9349379

rs11723436

rs4593108

rs17087335

rs2487928

rs7623687

rs11099493

rs56289821

rs16986953

rs10818576

rs12897

rs2107595

rs12826942

rs34232196

rs2552527

rs194937

rs9532984

rs12202017

rs9486719

rs115654617

rs1873197

rs2891168

rs35879803

rs1870634

rs2153219

rs116922558

rs9970807

rs288187

rs4468572

rs17678683

rs143803699

rs7212798

rs10857147

rs1199338

rs10840293

rs2128739

rs11065979

rs35895680

rs8068844

rs1412444

rs7500448

rs28451064

rs11556924

rs56336142

rs763475

rs55942719

rs2843152

rs748431

rs9914266

rs6909752

-1.0

-0.5

0.0

0.5

1.0

1.5

MR effect size for

'|| id:ieu-a-7' on 'Cholelithiasis || id:finn-b-K11_CHOLELITH'

**Figure S15**


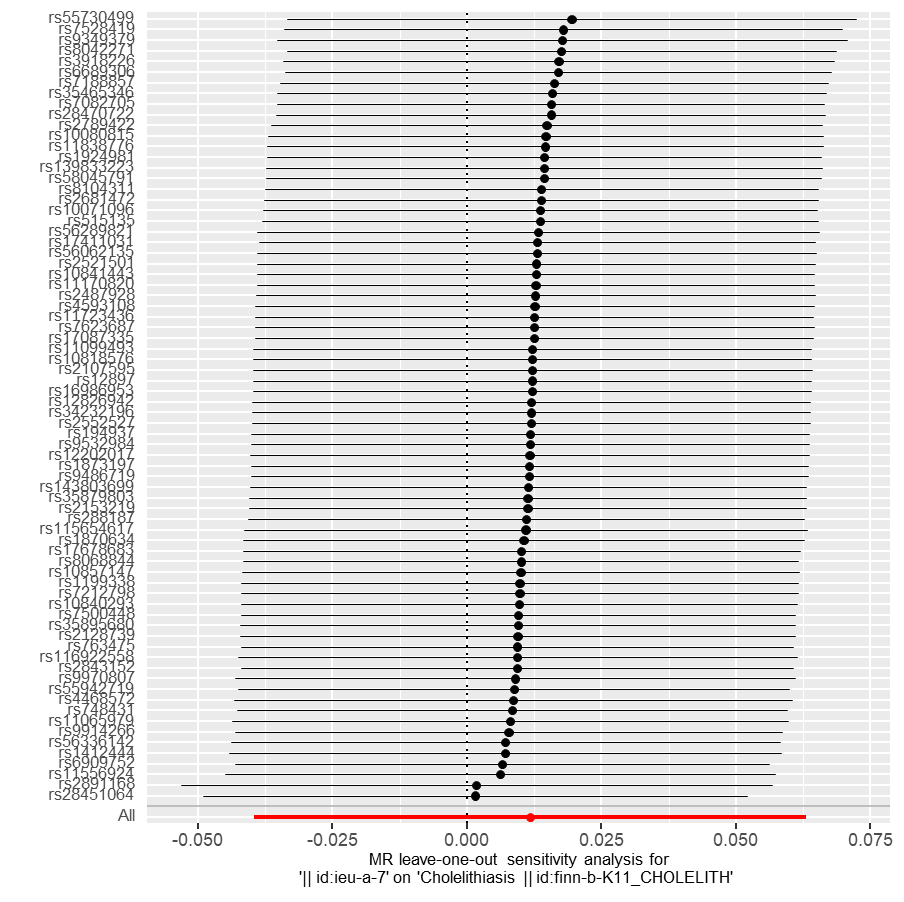


**Figure S16**


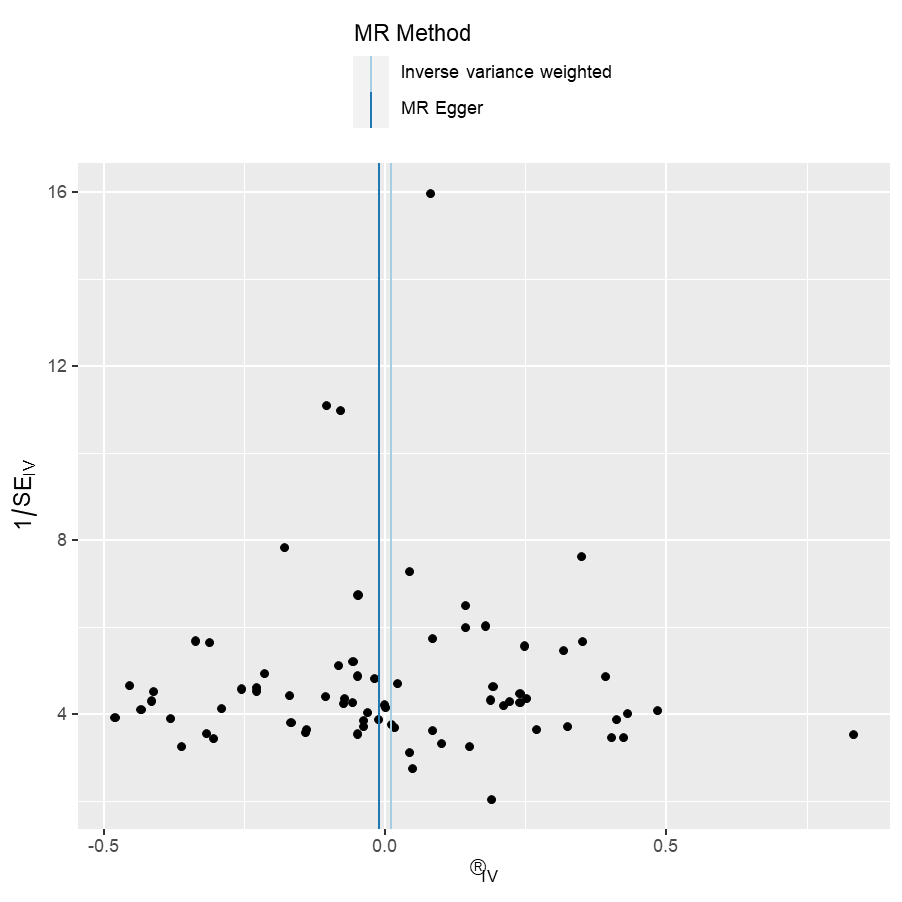


**Figure S17**


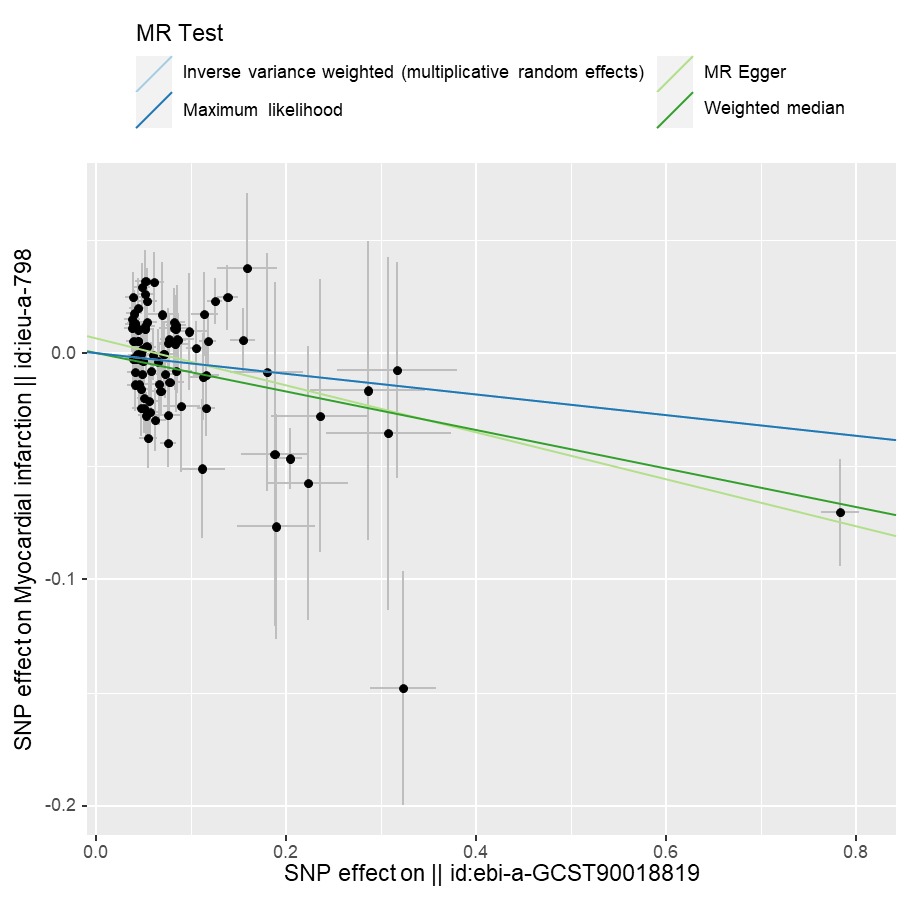


**Figure S18**


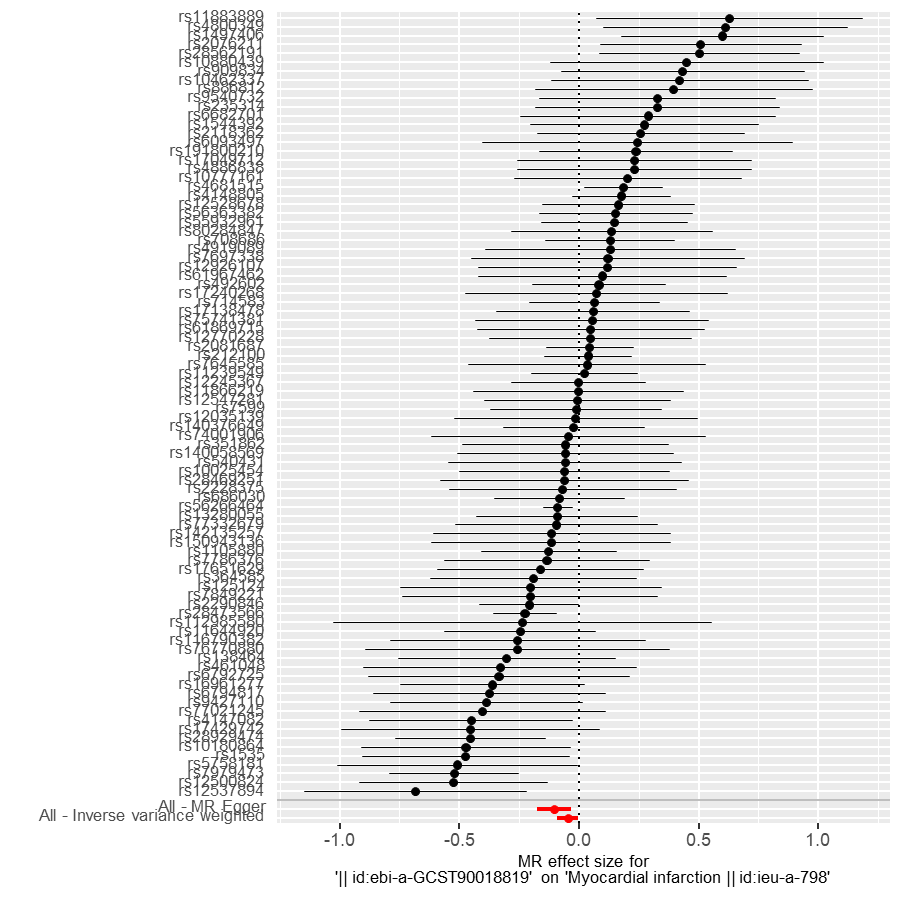


**Figure S19**


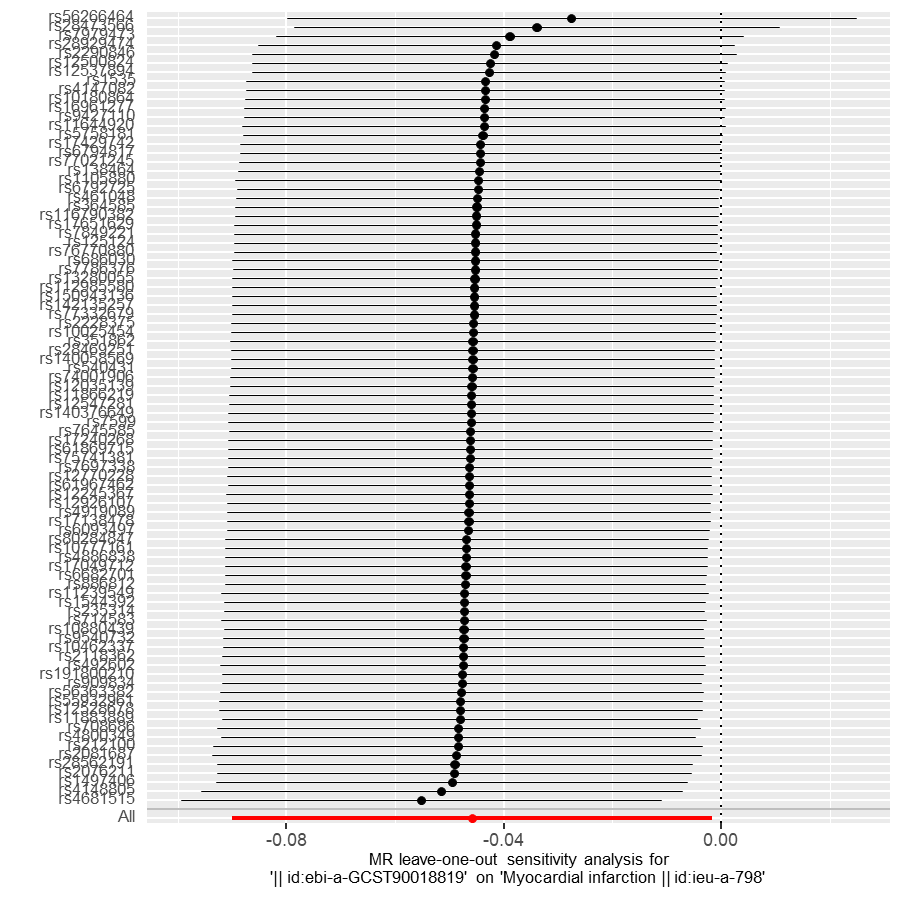


**Figure S20**


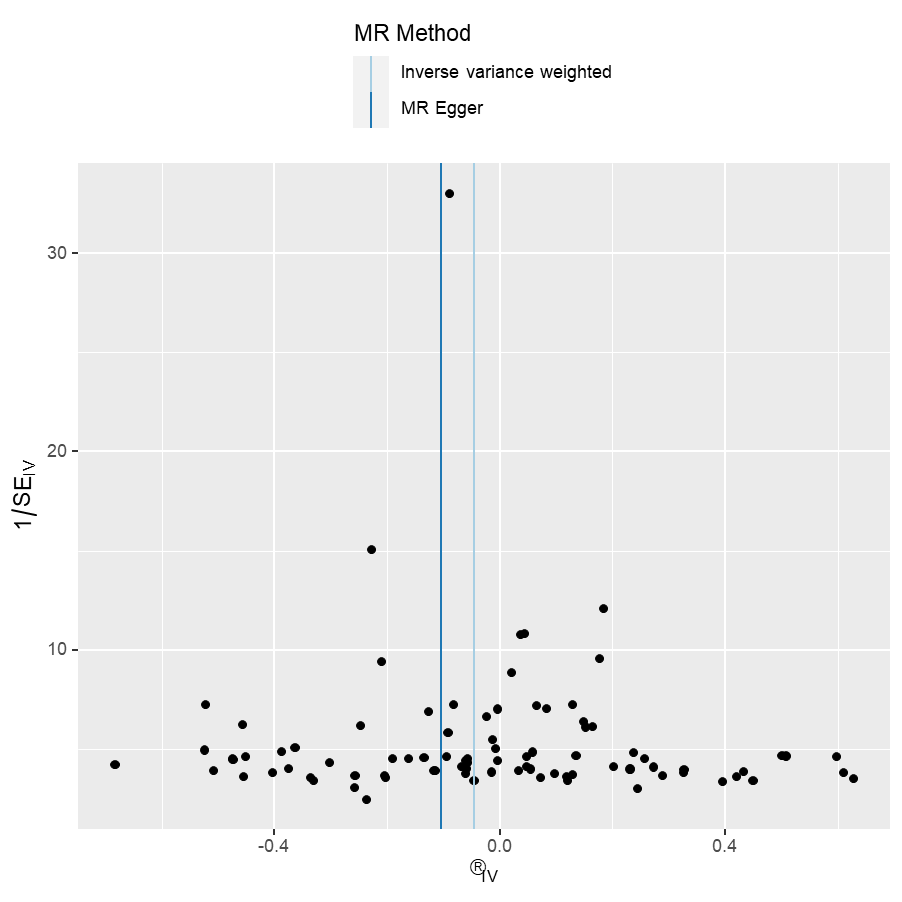


**Figure S21**


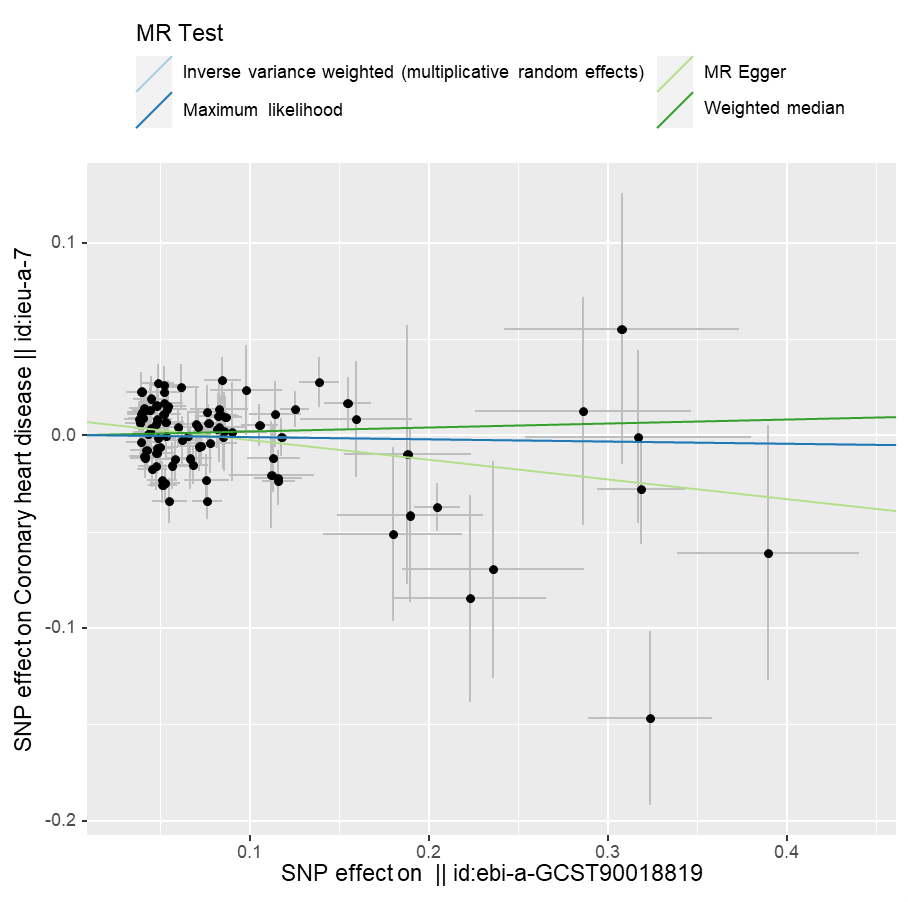


**Figure S22**


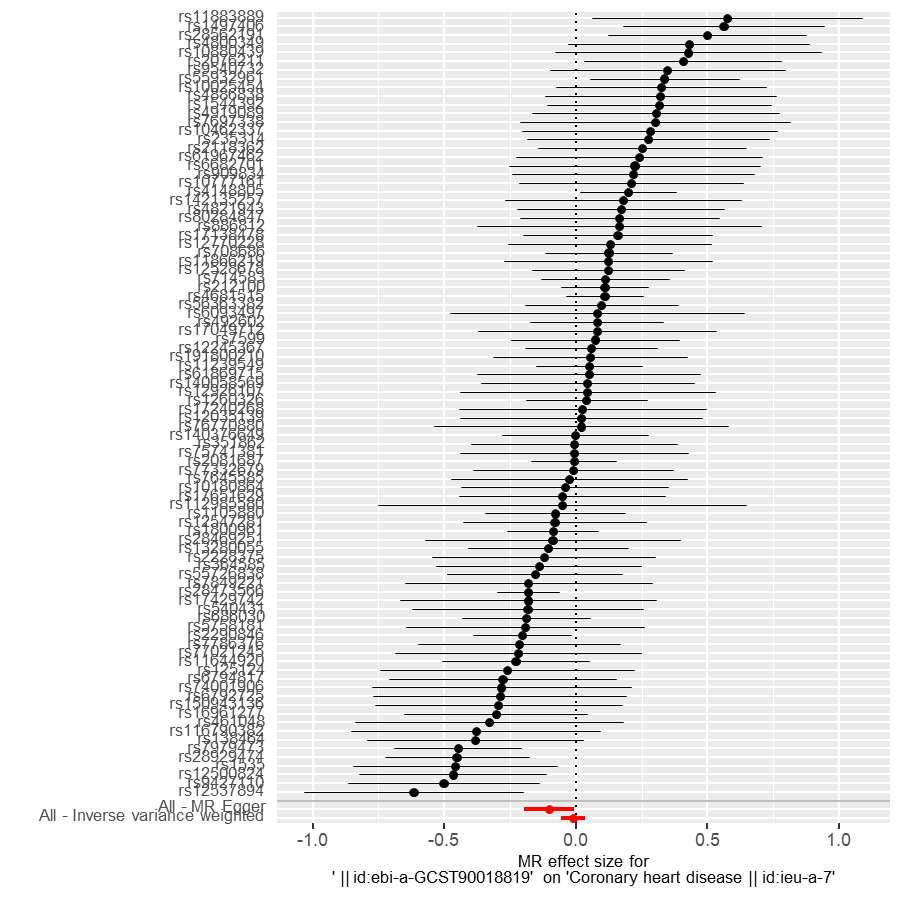


**Figure S23**


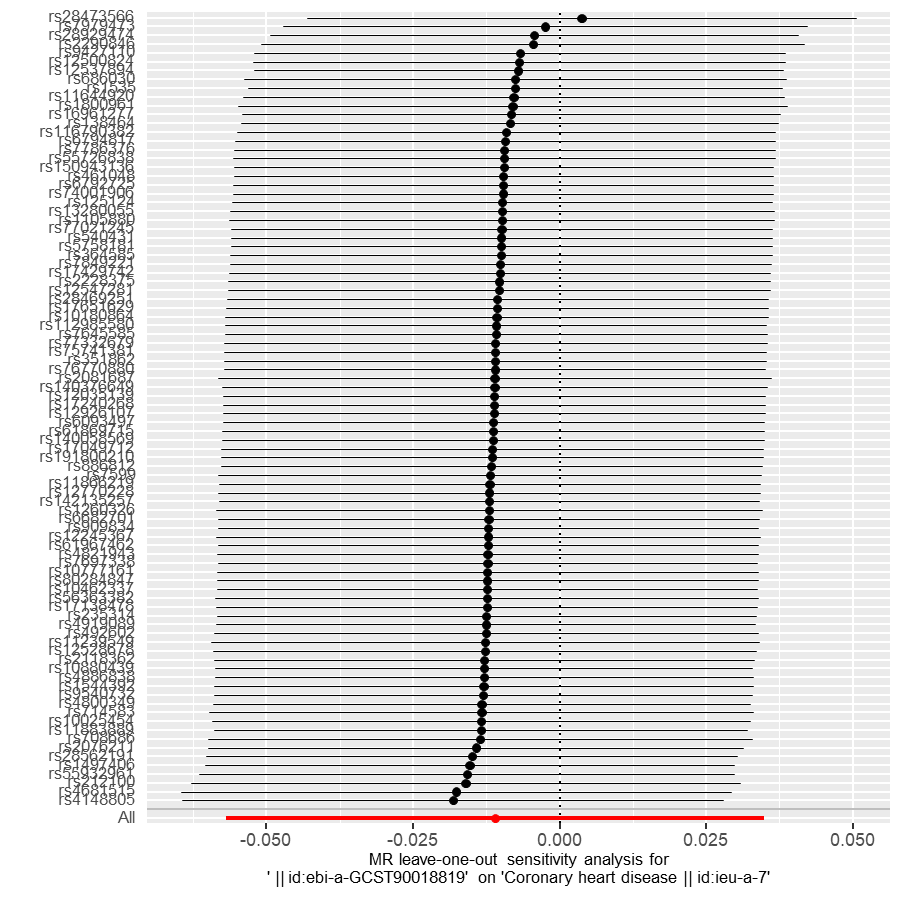


**Figure S24**


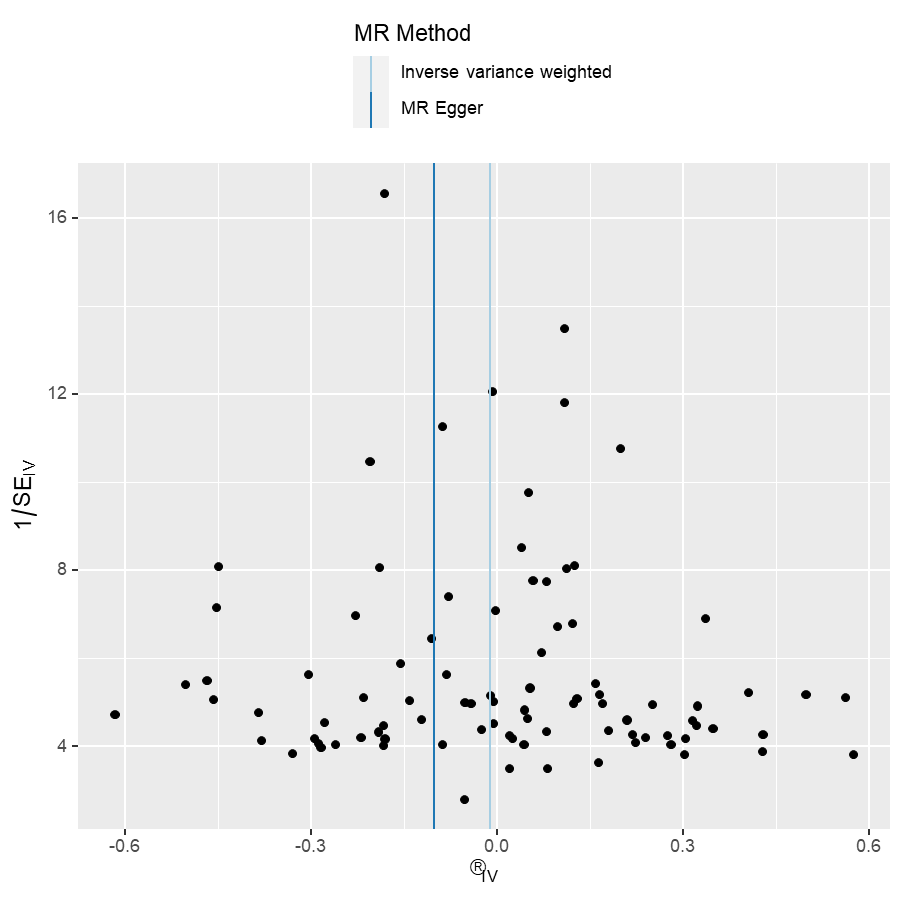

Supplement: Supplementary file 1 — Supplementary Figures. [file 41598_2023_46117_MOESM1_ESM.docx]
